# What Potential Authors Should Know About Publishing in *Global Health: Science and Practice*

**DOI:** 10.9745/GHSP-D-21-00540

**Published:** 2021-09-30

**Authors:** Stephen Hodgins, Sonia Abraham

**Affiliations:** a Editor-in-Chief, Global Health: Science and Practice Journal; Associate Professor, School of Public Health, University of Alberta, Edmonton, Alberta, Canada.; b Scientific Editor, Global Health: Science and Practice Journal, Baltimore, MD, USA.

In 2013, the U.S. Agency for International Development (USAID) and the Johns Hopkins Center for Communication Programs launched *Global Health: Science and Practice* (GHSP), responding to what we saw as an important gap in access to robust program-related evidence in global health. It was our conviction that a new kind of journal^1^ could both bridge that gap and better serve those engaged directly in program work. Our vision was to contribute to stronger, more effective programs and policies, helping to achieve greater population health impact.^2^ It has also been our ambition to exercise influence on global health-related publishing more broadly, shifting norms on inquiry into and documentation of program implementation issues, ultimately to better inform global health policy and practice.

## The Science of Practice

The “practice” in GHSP’s name has remained a central focus. As with other peer-reviewed journals, we certainly have been striving to contribute to evidence-based practice. What is not so typical of journals in this field, but that has been a central preoccupation for us is *practice-based evidence*—lessons that can be drawn from actual programs, especially those implemented at scale.

For programs to be more effective and achieve greater impact, the various actors of the system that create and use practice-based evidence need to be better connected.^3^ Program developers and managers, policy makers, advocates, and researchers need to be in continuous dialog, learning from each other, engaged in work informed by what other actors bring to the table.

“Science” is also centrally important to us, in several ways. Robust evidence on the effectiveness of interventions is certainly of interest to us, so methodologic rigor is an important criterion in our review process. In that respect, GHSP is similar to many other journals. But at least equally important for us is the real-world *utility* of study findings.

Furthermore, we believe *there’s a science to practice*; good, critical, reflective practice requires that we bring rigor and objectivity to our program inquiry. We need to take program performance measurement seriously, be open to disconfirming evidence, and be ready to move in new directions if evidence points us there. We believe that those engaged in program work are continuously drawing useful lessons from their work and that rigorously assessing and documenting and then sharing these lessons can benefit others. We’re interested in drawing lessons from exemplary programs, but we’re equally interested in those that have struggled. We’re concerned with what works (and doesn’t work), under what conditions, to achieve impact at scale on a sustained basis.

There is rich learning to be had from things that don't work as well as we planned. We understand that the success of a program is almost invariably attributable not only to the intervention itself but to a complex and dynamic interaction between the intervention and the specific context in which it is delivered. At GHSP, we expect authors to provide sufficient detail on the setting or context to allow readers to understand the interplay between intervention and context so they can make better-informed judgments of transferability to their own work settings. An important part of what we do at GHSP is to provide a platform for sharing such insights.

## How Do We Understand Rigor at GHSP?

Certainly, experimental designs provide stronger evidence for causal inference, but we don’t believe in an evidence hierarchy that automatically places randomized controlled trials and meta-analytic systematic reviews at the pinnacle. There are other kinds of evidence and other ways of understanding rigor or robustness that may be equally relevant when we’re trying to understand what works in the real world.

We are not saying that rigor, as conventionally understood, is not important. What we may think of as research principles certainly apply to program-based evidence. Authors need to be thinking about measurement validity, representativeness, counterfactuals and comparators, confounding, appropriate analytic methods, and sound causal reasoning. Even in opinion pieces, such as viewpoints and commentaries, authors need to offer well-supported arguments.

## Promoting Local Voices and Perspectives

Although work is often done in low- and middle-income countries by researchers based in high-income countries, it’s problematic for us when we receive articles on which the authors are overwhelmingly from high-income countries. This is not only a matter of fairness and equity but also of validity. Articles reflecting only an external perspective neglect valuable insights into the realities and nuances of the local system that authors and actors in LMICs can provide.^4^ We want to ensure that in-country authors are engaged and meaningfully contributing to documenting and making sense of practice-based evidence.

Similarly, though we welcome papers from donor-funded, in-country projects, we are especially interested in articles that reflect the input and perspective of those working in ministries of health and other in-country institutions.

Over the past year, we have intentionally refocused efforts to ensure that the articles we publish adequately reflect in-country voice and perspective. Last year, we updated our editorial policies to reflect our efforts to ensure that articles reporting findings from specific countries have meaningful participation from in-country authors.

## Diversity, Equity, and Inclusion

Similarly, last year,^5^ we recognized a need for a greater breadth of perspective within our associate editorial team and editorial board and greater diversity in gender, race, ethnicity, and geography. Appointing Abdulmumin Saad as deputy editor-in-chief and recruiting Rajani Ved as an associate editor were among the first steps in bringing broader programmatic expertise and experience as well as diverse perspectives and insights to our editorial team.

We are continuing to identify new editorial board members who can bring valuable research and program experience to help inform GHSP’s vision in the future.

## Eliminating Barriers to Publishing

In the field of open-access peer-reviewed journals, GHSP has the luxury of publishing articles with no submission fees to authors because of our special funding arrangements with USAID. This allows us to eliminate financial barriers disproportionately affecting early-career professionals and individuals in low- and middle-income countries, as they seek to share learning.^6^ To ensure access for authors who are under-represented in peer-reviewed journals, we aim to continue to publish GHSP without fees.

We recognize that we still have much to do in realizing our mission to contribute to more effective health programs and greater population health impact. We invite readers, authors, and reviewers to work with us toward this end and invite your suggestions on how we can do a better job.
